# Dental treatment outcomes in Thai children treated for severe early-childhood caries under general anaesthesia and non-pharmacological behaviour management: a retrospective study

**DOI:** 10.1007/s40368-024-00887-6

**Published:** 2024-05-24

**Authors:** N. Pratyaprateep, V. Jirarattanasopha, A. Smutkeeree

**Affiliations:** Department of Pediatric Dentistry, Faculty of Dentistry, Mahidol University, 6 Yothi Rd, Ratchathewi, Bangkok, 10400 Thailand

**Keywords:** Severe early-childhood caries, Dental treatment outcomes, General anaesthesia, Non-pharmacological behaviour management

## Abstract

**Purpose:**

The aim of this study was to compare dental-treatment outcomes, oral-hygiene improvement, and patient co-operation during follow-up visits between children treated under general anaesthesia (GA) and non-pharmacological behaviour management (NP).

**Methods:**

This retrospective study reviewed the dental chart records of healthy patients less than 71-month-old with severe early childhood caries (S-ECC) from 2008 to 2020 with at least a 6-month follow-up. The demographical data, dental-treatment outcomes, oral-hygiene status, and patient behaviour at the follow-up visits were analysed by the Mann–Whitney U test, Pearson’s Chi-square, Fisher’s exact test, Friedman test, and Wilcoxon test with a significance level of 0.05.

**Results:**

This study included 210 GA cases and 210 age-matched control NP cases. The GA group had a significantly higher caries experience, lower patient co-operation, poorer oral hygiene, and higher number of complex dental treatment than the NP group at baseline (*p* < 0.001). The number of children who had incomplete dental treatment under non-pharmacological behaviour management was higher than the GA group. After treatment, the number of new carious teeth in the NP group was significantly higher than in the GA group only at the 6-month follow-up. However, there was no significant difference in treatment failure, oral-hygiene improvement, and patient behaviour between groups.

**Conclusion:**

Although patients in the GA group had higher dental and behaviour problems than the NP group, the overall dental-treatment outcomes, including oral hygiene and behaviour improvement, were not significantly different between groups Therefore, regular follow-up and preventive treatment in the maintenance phase are essential for children with severe early-childhood caries.

## Introduction

Dental caries is “a biofilm-mediated, diet modulated, multifactorial, non-communicable, dynamic disease resulting in net mineral loss of dental hard tissue”. The caries lesion develops as a consequence of biological, behavioural, psychosocial and environmental factors. Early childhood caries (ECC) is the early onset of dental caries in young children under the age of 6 which has an atypical caries pattern, specifically on the smooth surfaces of the upper anterior teeth (Machiulskiene et al. 2020). Severe early childhood caries (S-ECC) is defined as “any sign of smooth-surface caries in a child younger than three years of age, and from ages three through five, one or more cavitated, missing (due to caries), or filled smooth surfaces in primary maxillary anterior teeth or a decayed, missing or filled score of greater than or equal to four (age 3), greater than or equal to five (age 4) or greater than or equal to six (age 5)” (American Academy of Pediatric Dentistry 2020). The consequences of ECC often include a higher risk of new carious lesions in the primary and permanent dentitions, hospitalizations, emergency room visits, high treatment costs, and school absence. ECC also diminishes the ability to learn and impacts the children’s oral health-related quality of life. The national oral-health survey in Thailand revealed that although the caries prevalence amongst 5-year-old children declined from 80.6% in 2007 to 78.5% in 2012 and 75.6% in 2017, there is still a high prevalence of dental caries in Thai children (Urwannachotima et al. 2019).

According to the American Academy of Pediatric Dentistry guideline (2023), behaviour guidance can be divided into two groups, basic and advanced behaviour guidance using either non-pharmacological (NP) or pharmacological options. Basic behaviour guidance comprises the techniques that depend on the art of communication and psychological approaches. In contrast, advanced behaviour guidance relies on specific tools and pharmacological approaches, e.g. protective stabilisation, sedation, and general anaesthesia (GA). Despite improvements in children’s dental health and the development of alternative treatment modalities, many young children with S-ECC and limited cooperativity still require comprehensive oral rehabilitation under pharmacological management to provide good quality dental care (Acs et al. 2001). However, many parents in Thailand who cannot afford dental treatment under GA or are uncomfortable with the pharmacological management procedures and related complications still prefer comprehensive dental treatment with NP behaviour management.

Dental fear and anxiety have been stated as a frequent and significant problem in children and adolescents worldwide. A current systematic review reported that the pooled prevalence of dental anxiety in preschool children was 36.5%, which was higher than schoolchildren and adolescents (Grisolia et al. 2021). Behaviour guidance techniques have been used to alleviate anxiety, nurture a positive dental attitude, perform high-quality oral-health care, are safe, and provide effective dental treatment for young children (Stiger 2016). Although, a systematic review by Ciancetti et al. (2017) concluded that NP and pharmacological interventions were useful to treat children with dental fear, no systematic review considered the effect of both interventions on children with dental fear and anxiety. However, the latest AAPD Clinical Practice Guideline of non-pharmacological behaviour guidance for the paediatric dental patient stated that overall basic behaviour-guidance techniques demonstrated a trivial-to small effect on reducing a patient’s anxiety and behaviour improvement (Dhar et al. 2023). S-ECC is one of the most common reasons to refer the patient for completing their dental treatment under GA by general dental practitioners (Chen et al. 2017a, b; Clayton and Mackie 2003; Podesta and Watt 1996; Grant et al. 1998; Holt et al. 1992; Smallridge et al. 1990). Many studies have reported that definitive treatment under GA was more likely to result in positive outcomes for children due to less frequent complications from failed restorations. (Al-Eheideb and Herman 2004; Lin and Lin 2015; Khodadadi et al. 2018). Nevertheless, a current systematic review revealed that dental treatment under GA did not help to prevent the development of new caries lesions and the rate of repeated GA was between 0  to  31% with the mean interval between GA treatments of approximately 2 years (Sabbahi 2022).

There is some evidence comparing dental-treatment outcomes under GA and the NP approach in children. Baakdah et al. (2021) compared the frequencies of the use and the completeness of dental treatment between pharmacological and NP interventions in patients up to 18 years old. Their results demonstrated that approximately two-thirds of the patients were treated with NP intervention whilst one-third received pharmacological intervention. The patients treated with GA required more restorations, extractions, and treatment that involved pain than those who received NP interventions. However, only 55% of patients with NP intervention completed their dental treatment. Grindeford et al. (2018) compared the operative and preventive treatments given prior to dental GA between the children treated under GA and the control group who were not receiving GA. The results showed that children treated under GA had a significantly higher number of decayed teeth, restorations, and frequency of dental visits than the non-GA group. Moreover, 48% of the GA group did not receive preventive treatment and 65% had no behaviour management treatment. Zhou et al. (2017) reported the results of a 2-year follow-up after dental treatment in 2–4 year-old children treated under GA and passive restraint (Zhou et al. 2017). Their study revealed that the oral-health habits in both groups were significantly improved, whilst new caries, recurrent caries, and the survival time of teeth in children treated under GA were significantly better than in the passive restraint group. However, this study was published in Chinese, thus, the full article could not be accessed.

In Thailand, the dental management of young children with S-ECC who have unco-operative behaviour would be either under NP management or GA due to limited financial support, dental resources, and parental concerns regarding pharmacological management procedures, including complications. However, there is no report on the dental treatment outcomes between the two behaviour management strategies in Thai children with S-ECC. Therefore, the objectives of this study were to compare the dental-treatment outcomes, oral-hygiene improvement, and patient co-operation during follow-up visits between children treated under GA and NP from dental records during 2008–2020.

## Materials and methods

This retrospective study was approved by the Ethical Committee of the Faculty of Dentistry/Faculty of Pharmacy, Mahidol University Institution Review Board (ref. No. COA.No.MU-DT/PY-IRB 2021/054.0906).

## Sample of the study

The sample size was based on the results of a previous study using the standard deviation (SD) of decayed teeth of the patients treated by GA (Khodadadi et al. 2018) and with an estimate of the difference for patients treated using NP as 25% of the patients treated by GA at the time of follow-up. Therefore, the sample size of this study was 210 children treated by GA and a 210 matched control group treated by NP to detect a difference in proportion with a 0.05 two-sided significance level.

The study population was healthy paediatric dental patients under 6 years old who had S-ECC and received comprehensive dental treatment. The first group was patients treated under GA, and the matched control group by age were patients who received dental treatment with NP from 2008–2020. The dental records of children who had less than 6 months of follow-up, and incomplete dental-treatment record were excluded from the study.

## Data collection

The retrospective chart review was collected from the patient dental records of children with S-ECC who received comprehensive dental treatment under GA or NP from 2008 to 2020 at the Pediatric Dental clinic, Faculty of Dentistry, Mahidol University. The data collection comprised the patients’ demographical data; age, sex, residency area, the simplified oral hygiene index (OHI-S) using the Greene and Vermillion index (Greene and Vermillion 1964), the decayed-missing-filled surfaces/teeth in the primary dentition (dmfs/dmft), behaviour level using Frankl behaviour rating scores (Stigers 2016), type of dental treatment, the dental-treatment outcomes, oral hygiene index, patient’s follow-up attendance, and patient behaviour during the follow-up visits.

The GA was performed by an anaesthesiology team and paediatric dental residents in one visit under the supervision of a highly experienced paediatric dentist at the Faculty of Dentistry, Mahidol University, Bangkok, Thailand. Intraoral radiographs were done in the operating room after the patients were unconscious in all cases. Treatment planning, including the type of restoration, and restorative material selection, were designed after evaluating the tooth condition and radiographic findings. Restorations and pulpal treatment were done under rubber dam application.

The children treated by the NP were also treated by paediatric dental residents under the supervision of a highly experienced paediatric dentist of the Faculty of Dentistry, Mahidol University, Bangkok, Thailand. They received comprehensive dental treatment in a routine dental setting using the NP approach, both basic and protective stabilisation if needed. Treatment planning was designed for each patient according to their co-operation, tooth condition, radiographic findings, and parent’s expectations. The dental treatment with NP could be either minimally invasive techniques or traditional restorative care that was determined by the dentist based on the clinical situation and parental needs. Minimal invasive techniques, including interim therapeutic restorations, fluoride varnish and silver diamine fluoride, were used to defer definitive dental treatment when the parents preferred non-painful dental procedures. Local anaesthetic injection was used for some dental procedures, such as pulpal treatment, stainless-steel crown, dental extraction and some restorations.

The oral examination and caries-risk re-assessment of both GA and NP groups were done during recall visits. The GA patients were appointed for a 1-week follow-up after GA to assess the dental-treatment outcome and identify any GA complications. The GA and NP groups were recalled according to their caries-risk level. Dental prevention and dental treatment, including behaviour management selection, were provided based on the patient’s oral-health status and caries-risk level.

## Reliability and reproducibility

The dental chart records were evaluated by the same dentist. Before the dental chart records were assessed, 10% of the dental chart records were randomly reviewed to evaluate the intra-examiner reliability with a mean kappa value of 0.880. Moreover, the examiner validity was tested by calibrating with an expert with a mean kappa value of 0.825.

## Statistical analysis

The data were analysed using SPSS (Statistical Package for the Social Science) software programme (version 21.0. SPSS Inc, IBM, Armonk, New York, USA). Descriptive statistics were analysed and reported as frequencies and mean with SD. The differences in mean age, caries experience, treatment needs, and OHI-S were analysed by the Mann–Whitney U test, whereas the other demographical data were analysed by the Pearson’s Chi-square and the Fisher’s exact test.

The comparison of new carious teeth, treatment failure and patient’s co-operation between the GA and NP groups during the follow-up periods was analysed by the Pearson’s chi-squared and the Fisher’s exact test. The difference in OHI-S during the follow-up periods and between the two groups was analysed by the Friedman test and Wilcoxon test, respectively. The confidence level was set at 95%, and a p-value < 0.05 was considered statistically significant.

## Results

**Fig. 1:**
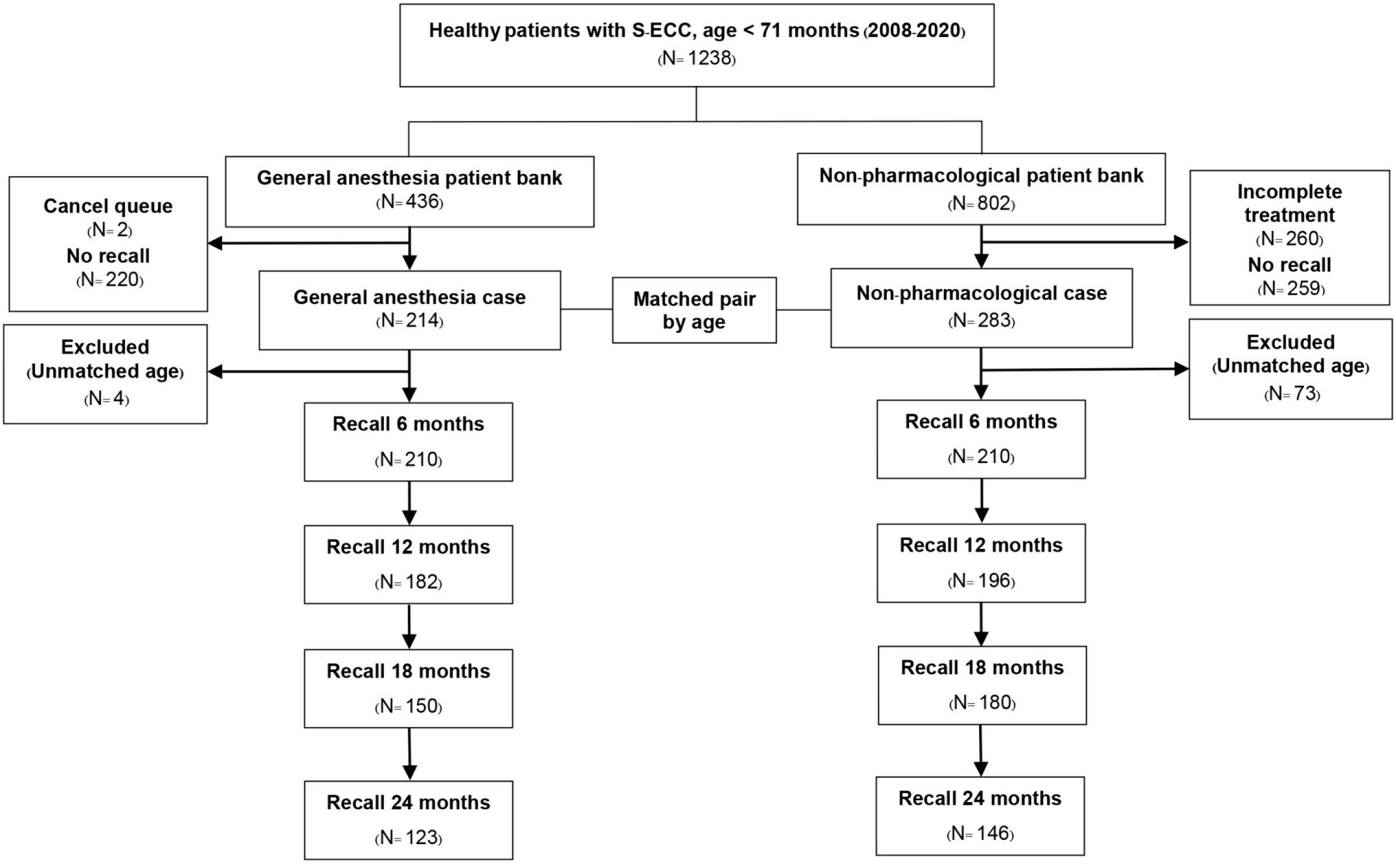
Case-recruitment flow chart

A total of 1238 dental chart records of healthy patients with S-ECC were reviewed, 260 were excluded due to incomplete comprehensive treatment, and 479 were excluded due to no recall visit. The age-matched control process was done, and 4 GA cases and 73 NP cases were randomly excluded due to unmatched age. Therefore, 420 cases were included in the study: 210 cases in the GA group and 210 cases in the NP group. By the 24-month follow-up, both groups experienced lost to follow-up and only 58.6% of the patients in the GA group and 69.5% of the patients in the NP group attended the follow-up appointments. The subject-recruitment flow chart in the study is shown in Fig. 1

## Demographical data

The mean age in the GA and NP groups were 39.53 $\pm$ 9.80 and 40.01 $\pm$ 10.77 months, respectively. There was no significant difference in age or sex between the groups (Table 1). The mean decayed, missing, filled teeth (dmft) and the mean decayed, missing, filled surfaces (dmfs) in the GA group were significantly greater than the NP group (*p* < 0.001), which indicated that the GA group had a higher caries experience than the NP group. There was a significantly higher number of cases with definitely negative behaviour in the GA group, whereas the NP group had a higher number of cases with positive behaviour (*p* < 0.001).

**Table 1:** Demographical data

	Total (*N* = 420)	General anaesthesia (*N* = 210)	Non-pharmacological (*N* = 210)	P-value
Age (months)				
Mean $\pm$ SD	39.77 $\pm$ 10.49	39.53 $\pm$ 9.80	40.01 $\pm$ 10.77	0.688
Range	20–70	21–69	20–70	
Sex (%)				
Male	220 (52.4)	116 (55.2)	104 (49.5)	0.241
Female	200 (47.6)	94 (44.8)	106 (50.5)	
Dmft				
Mean $\pm$ SD	10.78 $\pm$ 4.83	13.34 $\pm$ 4.04	8.22 $\pm$ 4.15	< 0.001^*^
Range	19 (1–20)	15 (5–20)	18 (1–19)	
Dmfs				
Mean $\pm$ SD	27.80 $\pm$ 18.99	36.79 $\pm$ 19.56	18.90 $\pm$ 13.41	< 0.001^*^
Range	97 (1–98)	92 (6–98)	58 (1–59)	
Frankl behaviour scores [Case (%)]				
Definitely negative (1)	188 (44.8)	142 (67.7)	46 (21.9)	< 0.001^*^
Negative (2)	177 (42.1)	61 (29.0)	116 (55.2)	
Positive (3)	53 (12.6)	7 (3.3)	46 (21.9)	
Definitely positive (4)	2 (0.5)	0 (0.0)	2 (1.0)	
Treatment needs				
Sealant				
Cases (%)	262 (62.4)	139 (66.2)	123 (58.6)	0.107
N/case	2.02 $\pm$ 2.08	2.08 $\pm$ 1.98	1.97 ± 2.29	0.423
Filling				
Cases (%)	375 (89.3)	185 (88.1)	190 (90.5)	0.430
N/case	3.90 ± 2.76	3.70 ± 2.74	4.10 ± 2.76	0.107
Stainless steel crown				
Cases (%)	325 (77.4)	207 (98.6)	118 (56.2)	< 0.001^*^
N/case	3.93 $\pm$ 3.49	6.14 $\pm$ 3.22	1.71 $\pm$ 2.05	< 0.001^*^
Pulp therapy				
Cases (%)	170 (40.5)	100 (47.6)	70 (33.3)	0.003^*^
N/case	0.66 ± 0.98	0.82 ± 1.10	0.50 ± 0.81	0.002^*^
Pulpotomy				
Cases (%)	52 (12.4)	39 (18.6)	13 (6.2)	< 0.001^*^
N/case	0.14 $\pm$ 0.41	0.22 $\pm$ 0.50	0.07 $\pm$ 0.50	< 0.001^*^
Pulpectomy				
Cases (%)	135 (32.1)	73 (34.8)	62 (29.5)	0.250
N/case	0.51 $\pm$ 0.89	0.60 $\pm$ 1.00	0.42 $\pm$ 0.75	0.159
Extraction (caries)				
Cases (%)	206 (49.0)	148 (70.5)	58 (27.6)	< 0.001^*^
N/case	1.74 $\pm$ 2.55	2.83 $\pm$ 3.00	0.65 $\pm$ 1.27	< 0.001^*^
OHI-S				
OHI-S index (mean $\pm$ SD)	1.80 $\pm$ 0.78	2.06 $\pm$ 0.77	1.54 $\pm$ 0.70	< 0.001^*^
OHI-S level [case (%)]				< 0.001^*^
Good (< 0.7)	19 (4.5)	5(2.5)	14 (6.7)	
Fair ( 0.7–1.7)	224 (53.3)	87 (41.5)	137 (65.2)	
Poor (1.8–3.0)	177 (42.2)	118 (56.0)	59 (28.1)	

*SD* standard deviation; *OHI*– S simplified oral hygiene index The differences in mean age, caries experience, treatment needs and OHI-S were analysed by the Mann–Whitney U test, whereas the other demographical data were analysed by the Pearson’s Chi-squared and the Fisher’s exact test. *Statistical significance (*P* < 0.05)

The number of complex dental treatments (stainless-steel crown, pulp therapy, and extraction) were significantly higher in the GA group than the NP group (*p* < 0.05). There was no significant difference in the number of sealants and restorations between groups. Moreover, there was a significantly higher mean OHI-S index in the GA group than the NP group (*p* < 0.001) and more than half of the GA group was categorised as a poor level of OHI-S index (Table 1).

## Dental treatment outcomes

Table 2 presents the new caries and treatment failure at the 6 – 18, and 24-month follow-up visits. The number of new dental caries cases, and the percentage of new carious teeth per the remaining teeth in the NP group were significantly greater than the GA group at 6 months. However, there was no significant difference between groups at the 12–18, and 24-month follow-ups. There was no significant difference in dental-treatment failure between the groups during all follow-up periods (*p* > 0.05).

**Table 2:** New caries and treatment failure during the follow-up periods

	6 Months			12 Months			18 Months			24 Months		
–	GA	NP	P-value	GA	NP	P-value	GA	NP	P-value	GA	NP	P-value
New caries	–	–	–	–	–	–	–	–	–	–	–	–
Cases (%)	51/210 (24.3)	110/210 (52.4)	<0.001^*^	67/182 (36.8)	89/196 (45.4)	0.09	59/150 (39.3)	85/180 (47.2)	0.15	45/123 (36.6)	62/146 (42.5)	0.326
No. of teeth/total (%)	122/1834 (6.6)	296/3297 (8.1)	<0.001^*^	155/1565 (9.9)	242/2906 (8.3)	0.25	115/1170 (9.8)	198/2477 (8.0)	0.175	85/916 (9.3)	150/1891 (7.9)	0.522
Treatment failure	–	–	–	–	–	–	–	–	–	–	–	–
[No. of teeth/total (%)]												
Sealant loss	27/410 (6.6)	26/414 (6.3)	0.304	12/360 (3.3)	22/361 (6.1)	0.392	27/298 (9.1)	17/297 (5.7)	0.373	19/220 (8.6)	19/224 (8.5)	0.823
Defective restoration	25/778 (3.2)	46/861 (12.7)	0.154	32/656 (4.9)	33/773 (4.3)	0.645	22/469 (4.7)	41/692 (5.9)	0.139	24/387 (6.2)	20/548 (3.6)	0.339
SSC dislodgement	8/1290 (0.6)	1/361 (0.3)	0.352	8/1045 (0.8)	1/341 (0.3)	0.16	5/912 (0.5)	3/322 (0.9)	0.231	2/761 (0.3)	0/249 (0.0)	0.515
Pulp therapy	2/172 (1.2)	6/105 (5.7)	0.284	14/154 (9.1)	6/94 (6.4)	1	10/107 (9.3)	2/82 (2.4)	1	6/76 (7.9)	1/66 (1.5)	1
Pulpotomy	2/46 (4.3)	1/15 (6.7)	1	7/42 (16.7)	1/14 (7.1)	0.127	4/26 (15.4)	1/13(7.7)	0.414	1/19 5.3)	1/11(9.1)	1
Pulpectomy	0/126 (0.0)	5/90 (5.5)	0.061	7/112 (6.2)	5/80 (6.2)	0.578	6/81 (7.4)	1/69(1.4)	0.05	5/57 (8.8)	0/55 (0.0)	0.09

*GA* general anaesthesia**,** *NP* non-pharmacological approach**,** *SSC* stainless-steel crown New caries and failure of treatments were analysed by the Pearson’s Chi-squared and the Fisher’s exact test Statistical significance (*P* < 0.05)

## Oral-hygiene status

The mean OHI-S of the GA group at baseline was significantly greater than the NP group (*p* < 0.001) (Table 3). The oral hygiene of all patients in the GA and NP groups was significantly improved at all follow-up periods compared with baseline. In the GA group, the oral hygiene index did not significantly change amongst the follow-up periods. In contrast, the oral hygiene index in the NP group gradually increased after the 12-month follow-up and the oral-hygiene status at the 6- and 12-month follow-ups were significantly better compared with the 18- and 24-month follow-ups. However, there was no significant change between the 6- and 12-month and 18- and 24-month follow-ups (Table 3).

**Table 3:** The *OHI*-*S* between baseline and follow-up periods in each group

OHI-S (mean $\pm$ SD)	General anaesthesia	Non-pharmacological	P-value
Before treatment (baseline)	2.06 $\pm$ 0.77^a^	1.54 $\pm$ 0.70^a^	< 0.001*
6 months	1.06 $\pm$ 0.64^b^	1.05 $\pm$ 0.61^b^	0.968
12 months	1.01 $\pm$ 0.60^b^	1.04 $\pm$ 0.63^b^	1.000
18 months	0.98 $\pm$ 0.53^b^	1.13 $\pm$ 0.63^c^	0.055
24 months	1.10 $\pm$ 0.67^b^	1.17 $\pm$ 0.59^c^	0.218
P-value	< 0.001^*^	< 0.001^*^	

*SD* standard deviation; *OHI*-*S* simplified oral hygiene index Comparison of *OHI*-*S *during follow-up periods were analysed by the Friedman test and comparison between groups were analysed by the Wilcoxon test *Statistical significance between groups (*P* < 0.05) Different superscript alphabet in each column indicates a significant difference amongst baseline and follow-up periods

## Patient co-operation

**Fig. 2:**
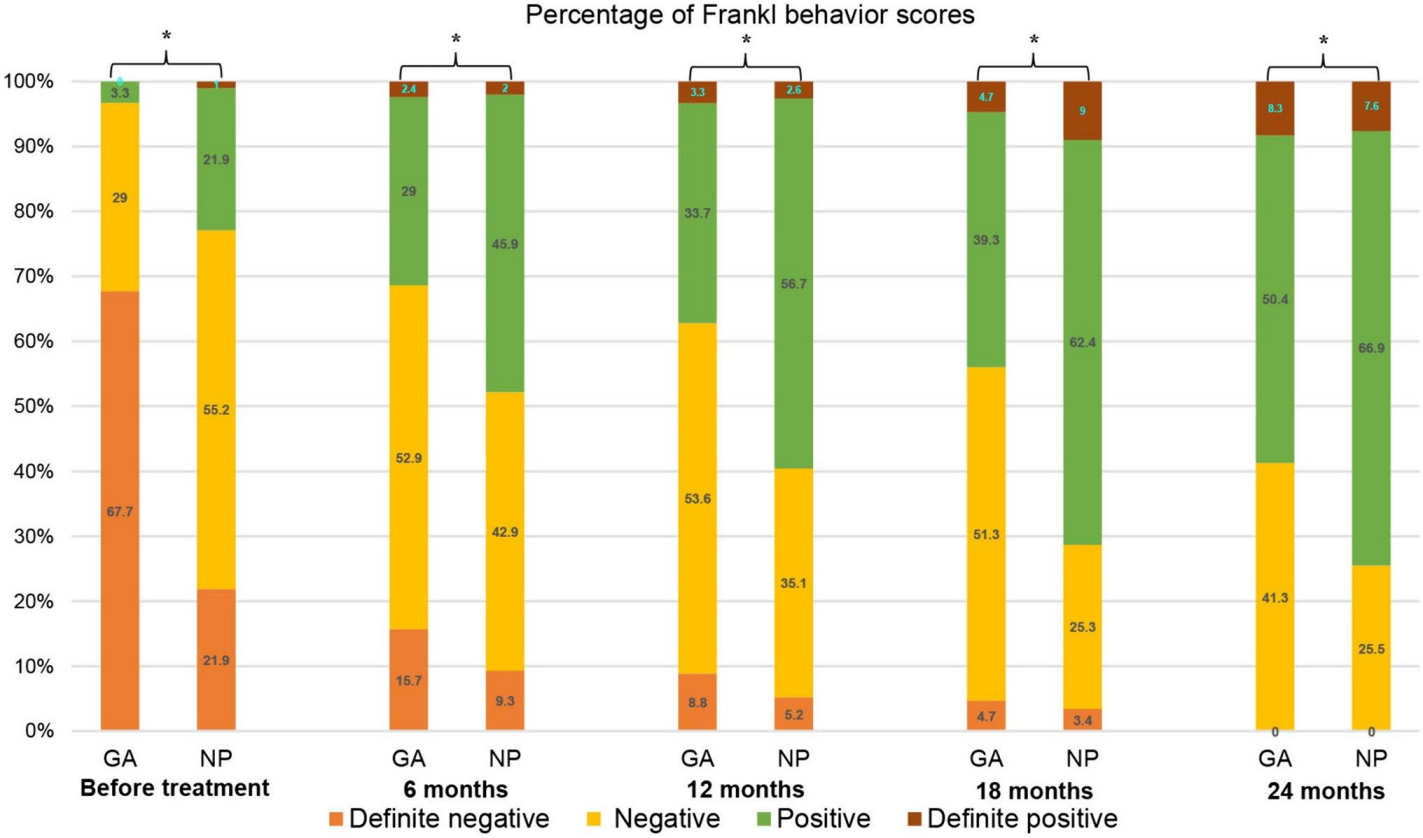
Frankl behaviour scores during the follow-up periods

The Frankl behaviour rating scale during the follow-up periods are presented in Fig. 2. The patients in the GA group demonstrated significantly better co-operation from baseline until the 24-month follow-up than the NP group patients. The GA and NP groups had a gradual improvement in co-operation during the follow-up periods.

## Discussion

The present study compared the dental-treatment outcomes, oral-hygiene improvement, and patient co-operation during follow-up visits between children treated under GA and NP from dental records during 2008 –2020. The results demonstrated that at baseline, the GA group had a significantly higher caries experience (dmft and dmfs), number of cases with definitive negative behaviour, and mean OHI-S than the NP group. The extensive dental treatment (e.g. stainless-steel crown, pulp therapy, and extraction) was significantly higher in the GA group than the NP group, which suggests that uncooperative children with extensive dental caries tended to receive dental treatment under GA. These finding were similar to those in Grindefjord (2018), who reported that children treated under GA had more decayed teeth and more restorations.

Dental treatment under GA is recommended as an effective behaviour management technique for unco-operative children with ECC, especially for children with extensive dental caries that requires extensive and multiple restorations without the need for their co-operation. Moreover, the number of complete cases was higher in patients treated by GA interventions (Baakdah et al. 2021) that was similar to the finding of the present study. However, the number of Thai children with S-ECC who received dental treatment under NP behaviour management was much higher than the GA group. This finding is possibly due to the limited facilities and government dental fee support in Thailand. Moreover, many parents of children with S-ECC are concerned about the pharmacological management procedures, related complications, financial issues, and long waiting time. This problem is possibly due to the multiple treatment visits required for patients who lacked psychological or emotional maturity. Therefore, this study used age-matched pairs between GA and NP groups to minimise the different outcomes and bias.

The result of this retrospective study revealed that ~ 70% of the children in both groups were maintained on a regular follow-up schedule. However, 6.7–18.9% of the patients in both groups dropped out from the follow-up appointment in each follow-up period (6, 12, 18, and 24 months). At the 24-month follow-up, there were 123 patients (58.5%) in the GA group and 146 patients (69.5%) in the NP group. There was no significant difference in outcomes between groups until the 24-month follow-up.

The percentage of new caries in the GA group was 6.6–9.9% within 6 − 24 months that was higher compared with Baseggio et al. (2010), who reported 3.13% new caries at the 24-month follow-up. However, our results were similar to a study by Jiang et al. (2019) that reported 8.5% and 18.8% of new carious cases at the 6-and 12-month follow-up, respectively. Although the preventive intervention was applied individually using the same standard guidelines in both GA and NP groups, the number of new carious teeth at the 6-month follow-up in the NP group was significantly greater than the GA group. This finding is explained by the different treatment planning between the NP and GA interventions. The NP group had more teeth prone to developing new caries than the GA group because many patients, whose parents preferred non-painful dental procedures, received minimal invasive techniques. In contrast, the treatment plans in the GA group was traditional restorations that contained a high number of painful dental procedures, such as a stainless-steel crown and extraction.

Furthermore, although the oral hygiene of both groups improved, the new caries in both groups were still quite high. These results indicated that Thai children with S-ECC have a high risk of dental caries. These results were supported by Boonyawong et al. (2022), who reported that 80.8% of preschool children had dental caries with a mean dmft of 8.2. They also found that older age, higher visible dental plaque score, and lower mother’s education level were significant risk factors for dental caries in preschool children. There was significant oral-hygiene improvement between before and after treatment in the GA group. Therefore, a caries-risk assessment and individual preventive approach during follow-up visits are necessary for preschool children.

We found that dental-treatment failure was not significantly different between the GA and NP groups from the 6–24-month follow-up. The restoration failure rate in this study ranged from 3.2 to 6.2% in the GA group and 3.6 − 12.7% in the NP group. More than 90% of the class II restorations in this study were restored with resin-modified glass ionomer cement (RMGIC). Previous studies demonstrated a wide range in the failure rate of class II restorations in primary posterior teeth with RMGI (2.58 – 16%) within 3 years in patients receiving NP treatment (Dermata et al. 2018; Webman et al. 2016). Furthermore, the composite and glass ionomer restoration failure rate under GA ranged from 9.9– 44.4% within 6 –24 months (Jiang et al. 2019; Biria et al. 2012). Therefore, the outcome of the dental restorations in this study was similar to those in previous studies.

The number of teeth with sealant loss within 24 months in the GA group was 3.3 –9.1% compared with the NP group at 5.7–8.5%. The rate of sealant loss in the GA group was similar to that in Jiang et al (2019) who reported a sealant loss of 7.1% at 6 months, and 10.7% at 12 months after being treated under GA. In the present study, the number of SSC dislodgements in both groups were low (less than 1%) compared with previous studies that reported a failure rate of SSCs placed under GA of 0.5% − 8% during the 6 − 36-month follow-up period (Jiang et al.^2019^; Biria et al. 2012; Al-Eheideb and Herman 2004; Tate et al. 2001; Sabbahi et al. 2022). In contrast, the failure rate of SSCs placed under NP in a previous study ranged from 2% − 25% during the 2 − 10-year follow-up periods (Randall 2002).

Pulp therapy was performed significantly more often in the GA group than the NP group, and pulpectomy was done more frequently than pulpotomy in both groups. The pulpotomy failure rate in this study (4.3% − 16.7%) was slightly higher than in previous studies that found a low-pulpotomy failure rate (1.1 – 14%) in teeth treated under GA within 6 −–36 months (Khodadadi et al. 2018; Biria et al. 2012; Al-Eheideb and Herman 2004; Sabbahi et al. 2022). However, the pulpotomy failure rate in this study was similar to other studies that reported a 1.9 –25.8% failure rate of pulpotomy with formocresol within 6 − 36 months (Trairatvorakul and Koothiratrakarn 2012; Dhar et al. 2017), and the pulpotomy with ferric sulphate failure rate was 15.4 –18.1% within 12 –24 months (Dhar et al. 2017). In contrast, the pulpectomy failure rate of in the present study (0– 7.4%) was lower than in previous studies that reported pulpectomy failure in the GA ranging from 0.8% − 9.5% in 6 − 33 months (Khodadadi et al. 2018; Jiang et al. 2019; Biria et al. 2012). However, it is difficult to compare the outcome of pulp treatment amongst different studies due to the variety of root canal materials used. Previous studies found that the pulpectomy failure rate in primary teeth with ZOE was 0% − 8% within 6 –18 months (Coll et al. 2020; Chen et al. 2017a,b ), and that of pulpectomy with Vitapex was 0% − 28.6% within 6 − 18 months (Chen et al. 2017b, a; Rasi et al. 2019; Trairatvorakul and Chunlasikaiwan 2008).

Both groups demonstrated improved patient co-operation during the follow-up visits. The NP group had significantly better co-operation than the GA group at all follow-up visits. Moreover, the GA group had a significantly twofold higher use of protective stabilisation during the follow-up visits (*p* = 0.001) (data not shown). These findings may be related to the baseline behaviour of children in the GA group who had more negative behaviour compared with the NP group. Furthermore, the children in the NP group received more behaviour management by dentists during treatment than the GA group who completed dental treatment in a single visit.

Although ECC treatment can be performed using GA and NP interventions, it has been reported that children with ECC had a poorer oral health-related quality of life compared with caries-free children (Singh et al. 2020). Therefore, providing prenatal oral-health care knowledge during pregnancy to prevent early acquisition of mutans streptococci, raising awareness of ECC with caregivers and health care professionals, limiting free-sugar consumption and daily fluoride exposure for young children are essential to prevent the development of ECC (Pitts et al. 2019; Xiao et al. 2019).

The limitation of this study was that some information could not be obtained from the dental chart records, such as the socioeconomic status of the caregivers. Moreover, the accuracy of retrospective data may not be as high as in a prospective study. Future prospective studies that evaluate dental-treatment outcomes are suggested.

## Conclusion

Considering the limitation of the present study, the following conclusions can be made:

- The children in the GA group had higher dental and behaviour problems compared with the NP behaviour management group.
- The number of children who had incomplete dental treatment in the NP behaviour management group was higher than in the GA group.
- There was no significant difference in dental-treatment outcomes, except for significantly more new carious teeth in the NP behaviour management group at the 6-month recall.
- Oral-hygiene improvement was observed in both groups after completing their dental treatment. However, there was no significant difference between the groups at all follow-up periods.
- The GA and NP behaviour management groups demonstrated behaviour improvement, however, the NP behaviour management group had significantly better co-operation than the GA group at all follow-up periods.

## Data Availability

Data available on request from the corresponding author.
